# Racial Differences in Functional and Sleep Outcomes with Positive Airway Pressure Treatment

**DOI:** 10.3390/diagnostics11122176

**Published:** 2021-11-23

**Authors:** Ikuyo Imayama, Bilgay Izci Balserak, Ahana Gupta, Tomas Munoz, Manassawee Srimoragot, Brendan T. Keenan, Samuel T. Kuna, Bharati Prasad

**Affiliations:** 1 Department of Medicine, Division of Pulmonary, Critical Care, Sleep and Allergy, University of Illinois at Chicago, Chicago, IL 60612, USA; iimaya2@uic.edu; 2 Department of Medicine, Jesse Brown VA Medical Center, Chicago, IL 60612, USA; 3 Department of Biobehavioral Health Science, University of Illinois at Chicago, Chicago, IL 60612, USA; bilgay@uic.edu; 4 Honors College, University of Illinois at Chicago, Chicago, IL 60607, USA; agupt47@uic.edu; 5 Department of Pediatrics, University of Illinois at Chicago, Chicago, IL 60612, USA; tmunoz@uic.edu; 6 College of Nursing, University of Illinois, Chicago, IL 60612, USA; msrimo2@uic.edu; 7 Department of Medicine, Division of Sleep Medicine, University of Pennsylvania, Philadelphia, PA 19104, USA; keenanbr@pennmedicine.upenn.edu (B.T.K.); Samuel.Kuna@va.gov (S.T.K.); 8 Department of Medicine, Corporal Michael J. Crescenz VA Medical Center, Philadelphia, PA 19104, USA

**Keywords:** obstructive sleep apnea, African American, race, functional outcome of sleep questionnaire, psychomotor vigilance task, actigraphy

## Abstract

It is unclear if the response to positive airway pressure (PAP) treatment is different between African American (AA) and European Americans (EA). We examined whether race modifies the effects of PAP on sleep and daytime function. We assessed Epworth Sleepiness Scale (ESS), Functional Outcomes of Sleep Questionnaire, Psychomotor Vigilance Task and actigraphy in 185 participants with moderate-to-severe obstructive sleep apnea before and 3–4 months after PAP treatment. The participants were middle-aged (mean, 55.1 years), 83.8% men and 60.5% AA. Linear regression models were used to examine the effect of race on outcomes. The AA had smaller reductions in ESS (mean change (95% confidence interval, CI) AA, −2.30 [−3.35, −1.25] vs. EA, −4.16 [−5.48, −2.84] and frequency of awakenings (AA, −0.73 [−4.92, 3.47] vs. EA, −9.35 [−15.20, −3.51]). A race × PAP usage interaction term was added to the model to examine if the change in outcomes per 1 h increase in PAP usage differed by race. AA exhibited greater improvement in wake after sleep onset (β (95% CI) AA, −8.89 [−16.40, −1.37] vs. EA, 2.49 [−4.15, 9.12]) and frequency of awakening (β (95% CI) AA, −2.59 [−4.44, −0.75] vs. EA, 1.71 [−1.08, 4.50]). The results indicate the importance of race in evaluating outcomes following PAP treatment.

## 1. Introduction

Obstructive sleep apnea (OSA) is characterized by episodes of repetitive obstruction of the upper airway [1] that are associated with oxyhemoglobin desaturations, sympathetic over-activation, inflammation and arousals [2]. These pathophysiological perturbations frequently manifest as disturbed sleep, daytime sleepiness, impairment in sustained attention and poor health-related quality of life [3,4,5,6]. Positive airway pressure (PAP) is the first-line treatment for OSA [7]. Multiple randomized trials and meta-analyses have demonstrated that PAP treatment improves daytime sleepiness and quality of life [8,9,10,11].

Epidemiological studies of OSA have shown that African Americans (AA) have a high prevalence of OSA [12,13,14]. Some studies showed an earlier onset [15] and greater severity of OSA in AA compared to European Americans (EA) [4,14,15,16,17,18]. AA race was associated with an approximately two-fold increased risk of severe OSA, hypertension and daytime sleepiness [14,17,18,19]. The socioeconomic and biological factors mediating these disparities remain unclear.

Despite the high risk of OSA and adverse health outcomes, AA race is associated with poor adherence to PAP therapy [20,21,22]. In addition, low socioeconomic status (SES) reduces PAP adherence [23,24]. The improvement in health outcomes after PAP treatment is partly determined by PAP adherence [7,25,26]. In the secondary analysis of a multicenter randomized controlled trial comparing home vs. laboratory based diagnosis of OSA and titration of PAP, AA race was a predictor of PAP adherence at 1 month after accounting for SES and severity of OSA [27]. AA race was not a predictor of PAP adherence at 3 months when SES was added as a covariate [27]. Further analysis of this study showed that sleep duration mediated the associations between black race and PAP adherence [28]. In a retrospective analysis of 227 veterans, PAP adherence was positively associated with functional outcomes scales (Functional Outcomes of Sleep Questionnaire, FOSQ) only in the AA group but not in EA or Hispanics group [29]. To our knowledge, there is little data on whether AA have a differential response to PAP treatment in terms of improvement in functional and sleep outcomes, independent of PAP adherence and SES.

The objective of this study was to examine whether AA race is associated with differential effects of PAP treatment on daytime sleepiness (Epworth Sleepiness Scale, ESS), functional outcomes (Functional Outcomes of Sleep Questionnaire, FOSQ), sustained attention (Psychomotor Vigilance Task, PVT) and sleep duration and quality measured by actigraphy. We hypothesized that AA with moderate to severe OSA would have an equivalent treatment response to PAP compared to EA after adjusting for PAP adherence and SES.

## 2. Materials and Methods

### 2.1. Participants and Study Design

The study participants were included from two cohort studies conducted at the Jesse Brown Veterans Affairs Medical Center (site 1) and the University of Pennsylvania (site 2). In brief, the site 1 study was a prospective study of 336 adults with moderate to severe OSA. The primary aim of the study was to examine the effects of PAP therapy on blood pressure outcomes (ClinicalTrial.gov NCT 01960465). The site 2 study was a prospective study with moderate to severe OSA performed in 2 sleep centers, University of Pennsylvania and University of Iceland. The primary goal was to compare the effects of PAP therapy on cardiovascular risk measures between obese and lean patients with OSA. Information on the study was previously published [30] (ClinicalTrial.gov NCT01578031). Study subjects from University of Pennsylvania were included in this analysis. Study subjects from University of Iceland were excluded due to differences in sociodemographic factors. The study inclusion criteria for both studies were adults with untreated OSA; apnea hypopnea index (AHI) ≥15/h by type 3 home sleep apnea test (HSAT) or polysomnography (PSG). Exclusion criteria were previous OSA treatment (PAP or oral appliance within six months or any surgical treatment), uncontrolled medical, psychiatric, or other sleep disorders (including shift work), substance or alcohol use and pregnancy. There were some differences in the inclusion and exclusion criteria and the study protocols between the two sites. In brief, the inclusion age range was 18 to 70 years for site 1 and 40 to 65 years for site 2, body mass index (BMI) >45 kg/m^2^ was an exclusion criterion for site 1 and BMI >40 kg/m^2^ for site 2, premenopausal women were excluded at site 2 and HSAT was used at site 1 and PSG at site 2 for evaluation of OSA. Participants other than AA or EA were excluded due to the small sample size (12% at site 1 and 6% at site 2).

After study consent was obtained, participants were tested for OSA using HSAT or PSG. HSAT (NightOne, Philips Respironics, Murrysville, PA, USA) was used at site 1 and attended PSG at site 2 (Sandman, Natus Medical, Middleton, WI, USA). The sleep tests were manually scored at each site by a certified scorer. HSAT recording was deemed acceptable with four or more hours of total recording time and at least 2 h of interpretable signals (oximetry, flow and effort). Similar American Association of Sleep Medicine specified scoring criteria were used for both types of tests. The 3% oxygen desaturation criterion (or arousals for PSG) was used for scoring hypopnea. After baseline measurements, those with AHI ≥15/h were treated with either fixed or auto-adjusting PAP and objective adherence data were downloaded from the devices (ResMed S9 or Airsense 10, ResMed, Inc. San Diego, CA, USA). The Institutional Review Board approved the protocol at each site. Research procedures were conducted and data was processed at each site individually. Some findings from site 2 have been previously reported [30].

### 2.2. Measurements

#### 2.2.1. Sample Characteristics

Demographic, self-identified race, active smoking, hypertension and diabetes and antihypertensive medication information was collected via questionnaires at the screening visit. We used the Center for Disease Control Social Vulnerability Index (SVI) to assess SES [31]. The SVI is a composite of four components: 1. Household composition and disability, 2. Socioeconomic status (income and education), 3. Minority status and language and 4. Housing type and transportation.

#### 2.2.2. Subjective Measures of Sleepiness and Functional Outcomes

The ESS was used to measure self-reported sleepiness [32]. The PVT 192 (Ambulatory Monitoring, Inc., Ardsley, NY, USA) was used to quantify deficits in sustained attention [33]. Both sites used a 10 min testing protocol in the morning. PVT median reaction time (RT) and number of lapses (errors of omission defined as RT >500 milliseconds), mean slowest RT and mean fastest RT were used as the sustained attention outcomes. The FOSQ was used for functional assessment [5].

#### 2.2.3. Objective Measures of Sleep

Quantitative sleep measures were obtained by wrist actigraphy recordings over 3 to 7 days (Actiwatch 2, Philips Respironics, Murrysville, PA, USA). The outcomes were measured at screening and final visit after 3 to 4 months. Wrist-worn actigraphy was recorded for 3–7 days at both sites using similar protocols (Actiwatch 2, Philips Respironics, Murrysville, PA, USA). The actigraphy had a sampling rate of 32 Hz. The data were downloaded and analyzed using Actiware software (version 5.71.0 or later) with a 30-s epoch. Sleep duration, wake after sleep onset (WASO) and frequency of awakening were extracted from actigraphy.

#### 2.2.4. PAP Device and Adherence Data

Auto-adjusting PAP was used at site 1 and either fixed or auto-adjusting PAP treatment (S9 or Airsense 10, ResMed, Inc., San Diego, CA, USA) was used at site 2. Fixed PAP pressure setting was determined by titration PSG. Participants treated with auto-adjusting PAP were started on a 4–20 cm H_2_O pressure range at both sites, which could be adjusted as clinically indicated. There were no restrictions regarding the type of mask interface and participants were allowed to change the size and model of their mask interface throughout the study. The research protocol did not include specific adherence interventions beyond standard clinical practice at either site. The adherence data were obtained from device downloads and the average use in hours per day across the treatment period of 3–4 months was used at both sites.

#### 2.2.5. Statistical Analyses

Subjects with AA or EA race and valid PAP adherence data were included in the analyses Baseline sociodemographic characteristics and outcomes among AA and EA groups were summarized using means and standard deviations or frequencies and percentages. Some PVT parameters were not normally distributed. The analyses were performed with both original and log plus 1 transformed variables for all PVT parameters. Because both analyses did not show significant differences, we have presented the result of original variables. Baseline comparisons were conducted using students *t*-test or Chi-square test as appropriate. To examine the effect of race (AA vs. EA) on change in outcomes, unadjusted and adjusted linear regression models were used (unadjusted analyses included AA vs. EA as a predictor, followed by adjustment for covariates: site, sex, age, body mass index, PAP adherence, AHI and SVI). The adjusted within-group mean (95% CI), as well as the between-group standardized mean differences (SMD) are presented in results. Absolute values of the SMD can be interpreted as small (0.2), moderate (0.5) or large (0.8).

Next, we examined the association of PAP adherence with outcomes using unadjusted and covariate (similar to variables noted above) adjusted linear regression models. To test whether this association differed between AA and EA, we conducted an interaction test evaluating the significance of the product term (PAP usage x Race) in a model that included the main effect plus the covariates listed above. A significant interaction suggests the slope of the association between PAP usage and outcome was different for AA vs. EA. Results are reported as adjusted β coefficients, where β is the expected mean change in outcome for a 1 h increase in PAP use. SAS software (version 9.4, Cary, NC, USA) was used for statistical analyses. A *p*-value of < 0.05 (2 tailed) was considered statistically significant.

## 3. Results

### 3.1. Sample Characteristics

**Figure 1:**
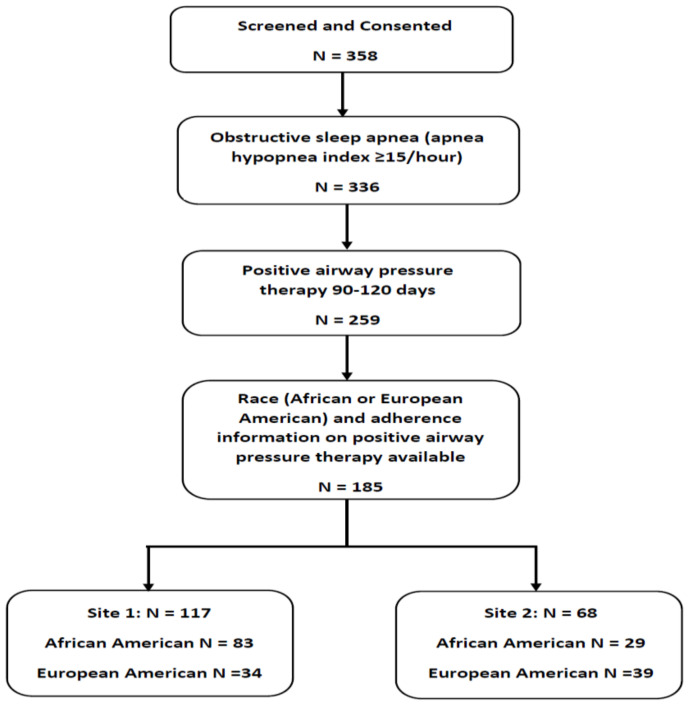
Flow diagram of study sample.

A total of 358 participants met eligibility criteria and were consented. 185 participants (51.7%) who completed the protocol and had PAP adherence data were included in the analyses (Figure 1). The study participants were middle-aged adults (age 55.1 ± 9.0 years), 83.8% were men and 60.5% were AA. There were several differences in baseline characteristics between the AA and EA groups (Table 1). Compared to the EA group, the AA group had more participants with hypertension and active smoking and a higher SVI score. All four components of the SVI were higher in AA vs. EA (*p* < 0.001 each for SES, household and disability and minority status and *p* = 0.0003 for housing and transportation), indicating an overall lower SES in AA. There were no differences in age, sex, BMI, or OSA severity measures. The AA group had lower adherence to PAP therapy than the EA group (2.79 ± 2.06 vs. 4.18 ± 2.28 h/day, *p* < 0.001). Other baseline difference between groups was a shorter total sleep time in AA (AA, 5.84 ± 1.40 vs. EA, 6.48 ± 1.19 h, *p* = 0.002, Table 2).

### 3.2. Group Comparisons of Subjective and Objective Sleep Outcomes

After CPAP treatment, the total sleep time per day increased in AA from an average of 5.84 h to 6.15 h with PAP, but remained lower than EA post-treatment (6.66 h, *p* = 0.078, Supplementary Table S1). The frequency of awakenings on actigraphy also remained higher in AA (34.9/h) than EA (29.3/h) post-treatment (*p* = 0.035).

Table 3 presents the change in outcomes within-groups and the SMD between-groups. Overall, both groups demonstrated significant improvements in ESS and total FOSQ after PAP treatment. While AA had significant improvements in all FOSQ subscales (general productivity, vigilance, social outcomes and sexual relations) except activity level, EA had improvements only in general productivity. Total daily sleep time by actigraphy did not change significantly in either group. WASO was reduced only in AA group (mean change (95% CI) = −14.87 [−28.92, −0.82], *p* = 0.038) and frequency of awakenings was reduced only in EA group (−9.35 [−15.20, −3.51], *p* = 0.002).

Between-group comparisons revealed that the AA group had evidence of smaller reductions in ESS score (mean change (95% CI] −2.30 [−3.35, −1.25] vs. −4.16 [−5.48, −2.84]) and frequency of awakenings (mean change (95% CI] −0.73 [−4.92, 3.47] vs. −9.35 [−15.20, −3.51]) after PAP treatment compared to EA, adjusting for covariates, including PAP adherence and SES measured by SVI (Table 3). Compared to EA group, SMDs for changes in ESS was (SMD (95% CI) = 0.35 [0.04, 0.66]; *p* = 0.046) and frequency of awakenings (SMD (95% CI) = 0.46 [0.10, 0.83]; *p* = 0.032) after PAP treatment in AA group.

### 3.3. PAP Responsiveness between Groups

**Figure 2:**
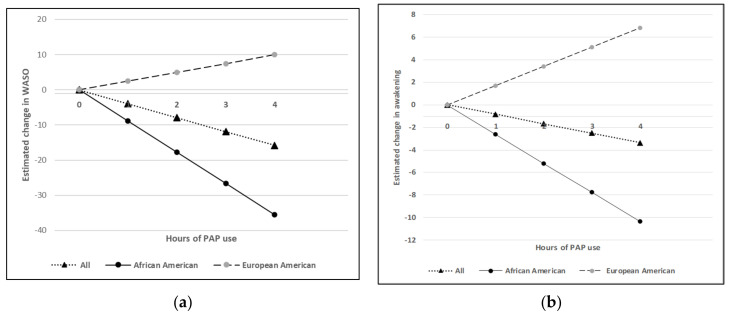
Estimated changes in outcome variables per 1 h increase in PAP use by each group: (**a**) Estimated changes in wake after sleep onset (WASO) per 1 h increase on positive airway pressure (PAP) use by each group; (**b**) Estimated changes in frequency of awakening per 1 h increase in PAP use by each group.

Next, we utilized statistical interaction tests to evaluate whether the change in outcomes for each 1 h increase in PAP usage differed based on race. There was evidence of significant interaction for changes in WASO (*p* = 0.003) and frequency of awakening (*p* = 0.0002), indicating that each 1 h increase in PAP usage led to greater reductions in WASO (β (95% CI) = −8.89 [−16.40, −1.37]; *p* = 0.021) and frequency of awakenings (−2.59 [−4.44, −0.75]; *p* = 0.007) among the AA group than in the EA group, which showed no significant relationship between PAP usage and change in either WASO (2.49 [−4.15, 9.12]; *p* = 0.452) or frequency of awakenings (1.71 [−1.08, 4.50]; *p* = 0.223) (Figure 2a,b). The interaction term was not significant for other outcomes, i.e., 1 h increase in PAP usage had similar degrees of treatment response in AA and EA.

Wake after sleep onset (WASO) and frequency of awakening, measured by actigraphy, demonstrated a significant difference in the response to increased PAP adherence based on race after adjusting for study site, sex, age, body mass index, apnea hypopnea index and social vulnerability index. As shown in Figure 2a, decrease in WASO per hour increase in PAP use was greater in African Americans (AA, β (95% CI) = −8.88 [−16.40, −1.36] vs. EA, 2.48 [−4.14, 9.12], *p* = 0.003 for Race ×PAP interaction). Similarly, Figure 2b demonstrates a significantly greater reduction in frequency of awakening in African Americans per hour increase in PAP use (AA, −2.59 [−4.44, −0.75] vs. EA, 1.71 [−1.08, 4.50], *p* = 0.0002 for Race × PAP interaction). The slopes presented in the figures were calculated based on the adjusted β for each group, relative to 0 h/night of PAP adherence.

## 4. Discussion

In this study, we report differences between AA and EA in functional and sleep outcomes after PAP treatment of moderate to severe OSA. This is one of the first studies to demonstrate that AA may have a different response to PAP treatment in terms of sleep outcomes independent of PAP adherence and SES. While the reductions in daytime sleepiness (ESS) after 3–4 months of PAP therapy were clinically meaningful, with a reduction of more than 2 points in each group [34], the overall reductions in ESS and sleep fragmentation (frequency of awakenings) were smaller in AA compared to EA independent of sex, age, SES, obesity, OSA severity and PAP adherence. Small to moderate differences based on race were observed for the changes with PAP for daytime sleepiness and sleep fragmentation (SMD of 0.35 and 0.46, respectively). However, a significant interaction between race and adherence indicated that increased PAP adherence resulted in greater improvements in WASO and frequency of awakening among AA compared to EA after adjustment for other potential predictors mentioned above. Our findings suggest that in moderate to severe OSA, PAP adherence promotion in AA is crucial and race-specific PAP adherence thresholds need to be considered for clinically meaningful improvements in functional and sleep outcomes. This will help bridge the racial disparities in OSA treatment and outcomes [35].

Improvement in OSA health outcomes is dependent on adherence to PAP therapy [7,25,26,36]. Similar to previous reports, we noted significantly lower PAP adherence among AA compared to EA [22]. Using data of a 3-month randomized controlled trial of 191 participants with moderate to severe OSA, Billings and colleagues showed that shorter sleep duration (<6 h) at baseline was associated with poor adherence and that short sleep duration mediated the association between AA race and low adherence [28]. In our study, the AA group had shorter total sleep time per day and smaller improvements frequency of awakening compared to EA group. Hence, the lower improvement in daytime sleepiness among AA in this study may be due to persistent sleep deficiency and interrupted sleep. Other potential mediators of PAP treatment effects on daytime sleepiness, such as comorbid medical and psychiatric disorders, should be investigated in future PAP intervention studies with sufficient samples of diverse racial groups.

Previous community-based studies have shown disparities in sleep duration, WASO and sleep fragmentation, with AAs experiencing poorer sleep on all measures [37,38]. The lower PAP effectiveness in improving sleep fragmentation in AA could be due to a higher burden of psychosocial stressors [39] or differences in the built environment [40,41]. Clinicians and researchers need to consider these differences in social and environmental factors when assessing response to PAP therapy in AA, determining the need for adjunctive treatments and designing adherence promotion interventions. Importantly, policymakers should consider these differences for setting patient-centered PAP adherence goals rather than a one-size-fits-all threshold of four hours per day [42].

To our knowledge, the interaction between race and PAP usage has not been systematically examined in the context of functional and sleep outcomes in OSA. Despite comparatively smaller improvements in WASO and frequency of awakenings in this study among AA, we show that increasing PAP use is likely to have a significantly greater impact on reducing WASO and frequency of awakenings in this group, thus improving key objective sleep measures. Notably, the slope of the association between PAP usage and WASO or frequency of awakenings was not significant in EA. This may be due to baseline differences in sleep architecture or a ceiling effect in the EA group [43,44,45]. Regardless, these results conceptualize the anticipated gain in outcomes with PAP usage can differ by race and need to be further explored. If confirmed, it will lead to the development of targeted treatment goals and outcome-specific adherence threshold recommendations for each racial group.

The overall FOSQ change in AA and EA was less than the minimum clinically important difference threshold of 1.8 [46]. This is not surprising given the low PAP adherence and higher baseline FOSQ of 15 compared to previous studies reporting values less than 13 in untreated OSA patients [5]. Similarly, the subscales did not show significant differences with PAP treatment in either group or between groups. These findings underscore the previous recommendations that 7 h nightly PAP use is required to attain an improvement in functional status [36]. This adherence threshold likely varies by OSA severity and baseline functional status, among other factors [47].

The number of PVT lapses in AA at baseline was numerically, though not statistically, higher than the EA group and met the threshold for sustained attention impairment in sleep deficiency states at 5 or more lapses [48,49]. No significant improvement in PVT median RT or number of lapses were noted in either group. PAP adherence has been reported to be positively associated with a reduction in PVT lapses, in a predominantly male population with moderate to severe OSA and daytime sleepiness [50]. The average PAP adherence in this group of patients was 3.3 h daily. Another study suggests that PAP use of 4 h per day, on sixty percent of days, improves PVT lapses only in severe OSA [51]. It remains unclear if there is a threshold of PAP adherence that predicts improvement in sustained attention. This should be examined in future studies that include diverse populations and account for other patient characteristics such as OSA severity, comorbid sleep disorders and habitual sleep duration.

The strengths of this study are the use of validated functional and objective sleep measures. Low SES is a strong predictor of poor PAP adherence [42,52] and we adjusted for both objective PAP adherence and SES using a comprehensive composite SES indicator, the SVI. This study is limited by using a combination of data from two different studies and significant participant drop-outs. Despite similar study design across the cohorts and adjustment for the site in multivariable models, unrecognized bias may persist. Similar to most PAP intervention research, the participants in this study had a low overall PAP adherence that limits outcome assessments. This study also did not include a quantitative measure of physical activity, potentially important predictor of sleep and functional outcomes in OSA. Nevertheless, this study provides novel insights into the differences between AA and EA in functional and sleep outcomes with PAP therapy.

## 5. Conclusions

In summary, we report that African Americans demonstrate less improvements in daytime sleepiness and sleep fragmentation than European Americans after PAP treatment of moderate to severe OSA. In contrast, African Americans showed evidence of greater benefits in objective sleep parameters with increased PAP usage. The results underscore the importance of optimizing PAP adherence and setting specific adherence targets based on the desired outcome and patient characteristics, including race. Future studies should define specific adherence targets and examine the factors mediating the racial differences in patient-centered functional and sleep outcomes with PAP treatment.

## Figures and Tables

**Table 1:** Baseline characteristics.

	N	African American	N	European American	*p*-Value
Demographics/Medical Conditions					
Age, years	112	54.7 ± 9.1	73	55.9 ± 8.9	0.354
Sex, % men	96	85.7	59	80.8	0.378
* Social vulnerability index	106	0.68 ± 0.24	67	0.42 ± 0.28	<0.001
Socioeconomic status (income and education)	106	0.71 ± 0.23	67	0.43 ± 0.29	<0.001
Household composition & disability	106	0.62 ± 0.27	67	0.43 ± 0.27	<0.001
Minority status & language	106	0.67 ± 0.17	67	0.53 ± 0.24	<0.001
Housing type & transportation	106	0.52 ± 0.26	67	0.40 ± 0.25	0.003
Body mass index, kg/m^2^	112	34.0 ± 5.3	73	33.6 ± 5.7	0.640
Hypertension, %	112	62	71	48	0.002
Diabetes, %	112	21	73	22	0.937
Current Smoker, %	111	31	72	17	0.033
Sleep testing					
Apnea hypopnea index, per hour	110	33.4 ± 18.6	72	34.2 ± 17.7	0.769
Time < SpO_2_ 90%, minutes	103	6.08 ± 8.04	62	7.63 ± 9.73	0.293
Oxygen desaturation index 3%, per hour	101	30.3 ± 19.9	55	28.8 ± 19.8	0.646
Treatment adherence					
Positive airway device usage, hours/day	112	2.79 ± 2.06	73	4.18 ± 2.28	<0.001

Data are presented as mean ± standard deviation or percentage. Chi-square or student *t*-tests were used to compare the differences between-groups. * Social vulnerability index—higher score indicates lower socioeconomic status.

**Table 2:** Comparison of outcome variables between African American and European American groups at baseline.

	Baseline				
	N	African American	N	European American	*p*-Value
Subjective outcomes					
Epworth Sleepiness Scale	110	11.8 ± 5.1	73	11.1 ± 5.6	0.376
Functional Outcome Sleep	111	15.1 ± 3.6	72	15.5 ± 3.4	0.524
Questionniare-10					
General productivity	109	2.97 ± 0.85	72	3.02 ± 0.83	0.679
Vigilance	110	2.99 ± 0.74	72	3.18 ± 0.72	0.083
Social outcomes	110	3.05 ± 0.98	72	3.29 ± 1.00	0.104
Activity level	110	3.28 ± 0.78	72	3.13 ± 0.74	0.177
Sexual relationships	109	2.86 ± 1.22	70	2.80 ± 1.22	0.739
Actigraphy					
Total sleep time, hours	99	5.84 ± 1.40	66	6.48 ± 1.19	0.002
Wake after sleep onset, min	100	72.1 ± 75.1	66	62.2 ± 35.4	0.260
Frequency of awakening, per hour	99	35.8 ± 14.0	66	33.4 ± 20.7	0.410
Psychomotor vigilance test					
Number of lapses	98	4.87 ± 8.04	72	4.24 ± 9.31	0.648
Median reaction time, msec	98	271 ± 59	72	264 ± 64	0.501
Mean slowest reaction time, msec	98	2.28 ± 0.65	72	2.43 ± 0.65	0.127
Mean fastest reaction time, msec	83	208 ± 38	70	202 ± 30	0.328

Data are presented as mean ± standard deviation.

**Table 3:** Comparison of changes in outcomes after 3–4 months of PAP treatment.

	Within Group Changes				Standardized Mean Difference	
	N	African American (AA)	N	European American (EA)	AA vs. EA	*p*-Value
Subjective outcomes						
Epworth Sleepiness Scale	98	−2.30 (−3.35, −1.25) **	66	−4.16 (−5.48, −2.84) **	0.35 (0.04, 0.66)	0.046
Functional Outcome	100	1.12 (0.38, 1.86) **	65	1.97 (1.02, 2.93) **	−0.17 (−0.49, 0.14)	0.197
Questionniare-10						
General productivity	98	0.28 (0.11, 0.46) **	64	0.40 (0.17, 0.62) **	−0.12 (−0.43, 0.20)	0.474
Vigilance	99	0.27 (0.13, 0.42) **	64	0.13 (−0.05, 0.32)	0.14 (−0.18, 0.46)	0.296
Social outcomes	98	0.29 (0.11, 0.47) **	62	0.12 (−0.11, 0.36)	0.22 (−0.10, 0.54)	0.319
Activity level	99	0.05 (−0.14, 0.24)	64	0.17 (−0.08, 0.42)	−0.19 (−0.51, 0.12)	0.505
Sexual relationships	96	0.36 (0.13, 0.59) **	59	0.30 (−0.01, 0.61)	−0.18 (−0.51, 0.14)	0.779
Actigraphy						
Total sleep time, hours	77	0.34 (−0.08, 0.76)	45	−0.24 (−0.82, 0.34)	0.11 (−0.26, 0.49)	0.143
Wake after sleep onset, min	78	−14.87 (−28.92, −0.82) *	45	2.01 (−17.59, 21.61)	−0.13 (−0.50, 0.24)	0.208
Frequency of awakening, per hour	78	−0.73 (−4.92, 3.47)	45	−9.35 (−15.20, −3.51) **	0.46 (0.10, 0.83)	0.032
Psychomotor vigilance test						
Number of lapses	72	−0.06 (−2.69, 2.58)	54	0.03 (−3.08, 3.13)	0.07 (−0.28, 0.43)	0.970
Median reaction time, msec	72	3.46 (−10.79, 17.71)	53	−8.01 (−25.01, 8.98)	0.12 (−0.24, 0.48)	0.341
Mean slowest reaction time, msec	71	0.84 (0.66, 1.03)	53	0.81 (0.60, 1.03)	0.26 (−0.09, 0.62)	0.835
Mean fastest reaction time, msec	54	61.19 (52.03, 70.35)	49	55.95 (46.25, 65.64)	0.37 (−0.01, 0.76)	0.472

Within group change in outcomes are presented as mean (95% confidence interval), adjusted for study site, gender, age, body mass index, average positive airway pressure use, apnea hypopnea index and social vulnerability index. * *p* < 0.05, ** *p* < 0.01. Standardized Mean Difference (SMD) is the standardized difference in outcomes change in African American vs. European American adjusted for study site, gender, age, body mass index, average positive airway pressure use, apnea hypopnea index and social vulnerability index. Data are presented as difference in mean change (95% confidence interval). SMD can be interpreted as small (0.2), moderate (0.5) or large (0.8).

## Data Availability

The data used to support the findings of this study are included within the article.

## Supplementary Materials

The following are available online at https://www.mdpi.com/article/10.3390/diagnostics11122176/s1, Table S1: Comparison of changes in outcome variables after 3–4 months of PAP treatment (Unadjusted).
