# Supplementary material for: Racial Differences in Functional and Sleep Outcomes with Positive Airway Pressure Treatment
Source: Diagnostics (Basel). 2021 Nov 23;11(12):2176. doi: 10.3390/diagnostics11122176 (PMC8700434; doi:10.3390/diagnostics11122176)
Supplement: Supplementary file 1 [file diagnostics-11-02176-s001.zip › diagnostics-1455371-supplementary.pdf]

Table S1. Comparison of changes in outcome variables after 3-4 months of PAP treatment (Unadjusted).

	Follow up				Within Group Changes				Standardized Mean Difference	
	**N**	**AA**	**N**	**EA**	**N**	**AA**	**N**	**EA**	**AA vs. EA**	**P-value**
**Subjective outcomes**										
Epworth Sleepiness Scale	102	9.3 ±4.0	68	7.0 ± 4.5	100	-2.38 (-3.38, -1.38)	68	-4.21 (-5.42, -2.99)	**0.36 (0.05, 0.66)**	**0.023**
Functional Outcome Sleep Questionniare-10	102	16.2 ± 3.6	67	17.2 ± 2.9	101	1.21 (0.52, 1.89)	66	1.77 (0.92, 2.62)	-0.16 (-0.47, 0.15)	0.313
General productivity	101	3.22 ± 0.86	65	3.39 ± 0.70	100	0.28 (0.12, 0.43)	65	0.38 (0.18, 0.57)	-0.13 (-0.44, 0.19)	0.425
Vigilance	101	3.26 ± 0.77	65	3.37 ± 0.62	101	0.26 (0.12, 0.41)	65	0.22 (0.04, 0.40)	0.06 (-0.25, 0.38)	0.670
Social outcomes	100	3.33 ± 0.83	63	3.40 ± 0.85	100	0.31 (0.13, 0.49)	63	0.17 (-0.05, 0.40)	0.15 (-0.17, 0.47)	0.347
Activity level	101	3.34 ± 0.90	65	3.34 ± 0.84	101	0.04 (-0.13, 0.22)	65	0.22 (0.00, 0.44)	-0.20 (-0.51, 0.11)	0.210
Sexual relationships	98	3.07 ± 1.14	62	3.19 ± 1.19	98	0.27 (0.05, 0.48)	60	0.45 (0.18, 0.72)	-0.17 (-0.50, 0.15)	0.293
**Actigraphy**										
Total sleep time, hours	84	6.15 ± 1.65	51	6.66 ± 1.61	79	0.30 (-0.10, 0.70)	46	0.09 (-0.44, 0.62)	0.12 (-0.25, 0.48)	0.535
Wake after sleep onset, min	84	63.7 ± 41.2	51	64.1 ± 42.1	80	-11.42 (-23.99, 1.15)	46	-3.06 (-19.63, 13.52)	-0.15 (-0.51, 0.22)	0.427
Frequency of awakening, per hour	84	34.9 ± 15.8	51	29.3 ± 14.2	79	-1.18 (-5.05, 2.69)	46	-8.27 (-13.33, -3.20)	0.40 (-0.04, 0.76)	**0.030**
**Psychomotor Vigilance Tests**										
Number of lapses	85	5.33 ± 7.80	57	4.67 ± 6.70	75	0.41 (-1.90, 2.73)	56	-0.30 (-2.98, 2.37)	0.07 (-0.28, 0.42)	0.689
Median reaction time, msec	85	270 ± 56	56	267 ± 51	75	1.26 (-11.30, 13.82)	55	-5.90 (-20.56,8.77)	0.13 (-0.22, 0.48)	0.465
Mean slowest reaction time, msec	85	3.23 ± 0.97	56	3.05 ± 0.83	74	0.91 (0.71, 1.11)	55	0.69 (0.46, 0.92)	0.25 (-0.10, 0.60)	0.166
Mean fastest reaction time, msec	60	274 ± 65	51	252 ± 65	57	69.12 (54.27, 83.96)	51	46.96 (31.26, 62.65)	0.39 (0.01, 0.76)	0.044

AA African American, EA European American

Within group change in outcomes are presented as mean (95% confidence interval)

Standardized Mean Difference (SMD) is the standardized difference in outcomes change in African American vs. European American. Data are presented as difference in mean change (95% confidence interval). SMD can be interpreted as small (0.2), moderate (0.5) or large (0.8).
